# Finding Cortical Subregions Regarding the Dorsal Language Pathway Based on the Structural Connectivity

**DOI:** 10.3389/fnhum.2022.784340

**Published:** 2022-05-02

**Authors:** Young-Eun Hwang, Young-Bo Kim, Young-Don Son

**Affiliations:** ^1^Neuroscience Convergence Center, Korea University, Seoul, South Korea; ^2^Department of Health Sciences and Technology, Gachion Advanced Institute for Health Sciences & Technology (GAHIST), Gachon University, Incheon, South Korea; ^3^Department of Biomedical Engineering, Gachon University, Incheon, South Korea; ^4^Department of Neurosurgery, Gil Medical Center, College of Medicine, Gachon University, Incheon, South Korea

**Keywords:** diffusion-weighted magnetic resonance imaging (dMRI), language pathway, tractography, connectome, superior longitudinal fasciculus (SLF), arcuate fasciculus (AF)

## Abstract

Although the language-related fiber pathways in the human brain, such as the superior longitudinal fasciculus (SLF) and arcuate fasciculus (AF), are already well-known, understanding more sophisticated cortical regions connected by the fiber tracts is essential to scrutinize the structural connectivity of language circuits. With the regions of interest that were selected based on the Brainnetome atlas, the fiber orientation distribution estimation method for tractography was used to produce further elaborate connectivity information. The results indicated that both fiber bundles had two distinct connections with the prefrontal cortex (PFC). The SLF-II and dorsal AF are mainly connected to the rostrodorsal part of the inferior parietal cortex (IPC) and lateral part of the fusiform gyrus with the inferior frontal junction (IFJ), respectively. In contrast, the SLF-III and ventral AF were primarily linked to the anterior part of the supramarginal gyrus and superior part of the temporal cortex with the inferior frontal cortex, including the Broca's area. Moreover, the IFJ in the PFC, which has rarely been emphasized as a language-related subregion, also had the strongest connectivity with the previously known language-related subregions among the PFC; consequently, we proposed that these specific regions are interconnected *via* the SLF and AF within the PFC, IPC, and temporal cortex as language-related circuitry.

## Introduction

Language is considered one of the most compelling indications of human cognitive activity. With much reasonable evidence that language activities proceed over macroscale networks in the human brain (Dick and Tremblay, 2012), finding language pathways has attracted significant interest from many researchers. As structural connectivity studies of neuronal fiber pathways increase rapidly, language researchers are focusing on constructing a consistent and systematic language framework that has been consolidated with lesion studies (Schmahmann et al., 2008; Bernal and Ardila, 2009; Fridriksson et al., 2010; Dick and Tremblay, 2012). For example, abnormal or impaired fiber pathways may involve specific functional deficiency, so comprehending the language deficiency related to the specific lesion areas can offer crucial insight into clinical interaction. However, individual fiber bundles are difficult to identify, even though their names and functions have been studied separately. Above all, their spatial location, origin, and termination have been debated (Yamada, 2009; Brauer et al., 2011; Dick and Tremblay, 2012).

**Figure 1:**
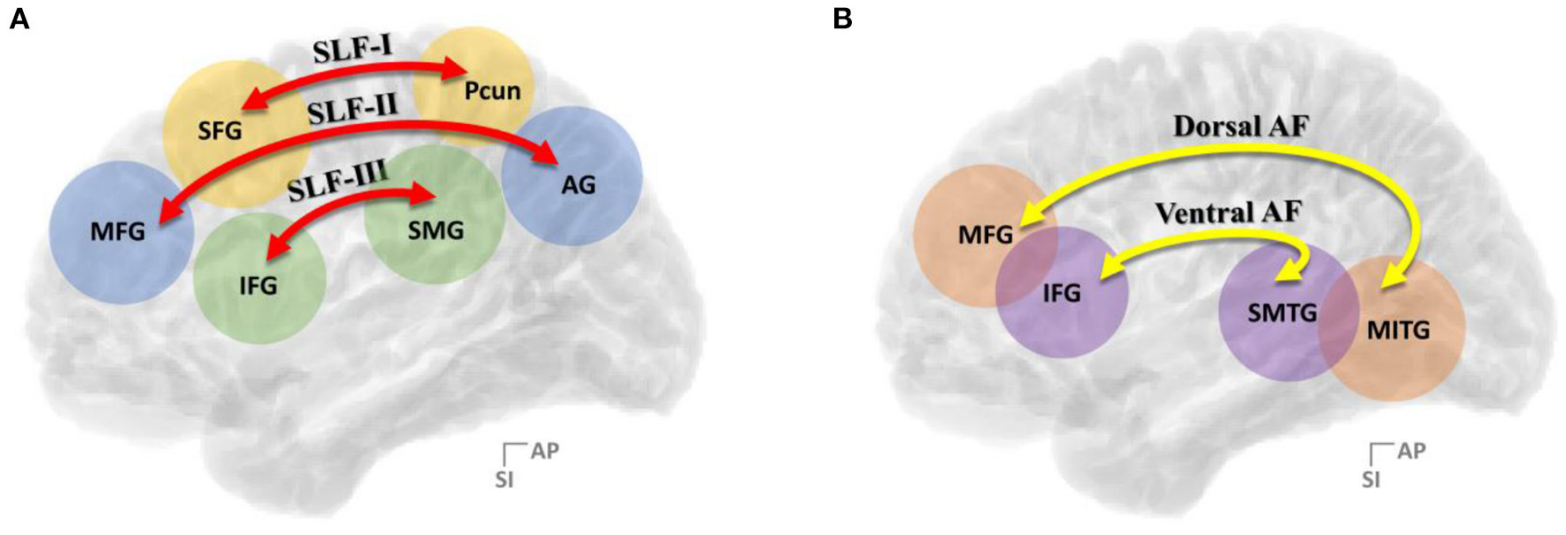
Summary of the **(A)** three-segment model of SLF connectivity and the **(B)** two-segment model of AF connectivity. SLF, superior longitudinal fasciculus; AF, arcuate fasciculus; AG, angular gyrus; IFG, inferior frontal gyrus; MFG, middle frontal gyrus; MITG, middle and inferior temporal gyri; SFG, superior frontal gyrus; Pcun, precuneus; SMG, supramarginal gyrus; SMTG, superior and middle temporal gyrus.

In the context of the contemporary dual stream model for language processing, the dorsal language stream controls phonological articulation and supports the mapping of lexical–semantic representations for word production, while the ventral language stream is involved in semantic processing and maps sensory inputs to meaning for comprehension (Saur et al., 2008; Chang et al., 2015; Fernández-Miranda et al., 2015; Yagmurlu et al., 2016; Ries et al., 2019). Among the well-known language pathways describing the dual-stream model for language processing, the dorsal language stream, the superior longitudinal fasciculus (SLF), and the arcuate fasciculus (AF) in the left hemisphere have been emphasized as the primary association fiber pathways crucial to language-related activities (Wernicke, 1908) and extensively studied over the past few decades. Especially, it has been still debated regarding its role on the semantic processing for language production and comprehension. These association fiber bundles that connect the temporoparietal regions with the frontal areas were considered indissociable fiber bundles previously (Dick and Tremblay, 2012) but were separated from each other in recent studies. Moreover, there have been various efforts to subdivide SLF and AF further in recent years (Figure 1). Accordingly, the SLF, which connects the frontal cortex to the parietal cortex, has been generally divided into three branches, namely, SLF-I, SLF-II, and SLF-III (Makris et al., 2005; Thiebaut et al., 2011; Kamali et al., 2014; Wang et al., 2016; Yagmurlu et al., 2016), while the AF, which connects the frontal cortex and temporal lobe, has been broadly divided into two branches, namely, dorsal AF and ventral AF (Glasser and Rilling, 2008; Yagmurlu et al., 2016). Specifically, SLF-II and SLF-III are the pathways that link the inferior parietal lobule, otherwise known as Geschwind's territory, with the frontal regions responsible for the articulatory aspects of language and visuospatial awareness (Saur et al., 2008; Brownsett and Wise, 2010; Fridriksson et al., 2010; O'Connor et al., 2010; Wang et al., 2016, 2017; Yagmurlu et al., 2016). In contrast, the dorsal and ventral AFs have been studied for their roles in phonological processing, which proceeds the receptive processing of phonemes and expressive production of phonemes during a speech, and lexical–semantic processing, which associates the vocabulary with its meaning (Poldrack et al., 1999; Keller et al., 2001; Glasser and Rilling, 2008; Roelofs, 2014; Fernández-Miranda et al., 2015; Yagmurlu et al., 2016). Moreover, even in terms of clinical correlation, the relative importance of the SLF and AF has been neglected compared with their cortical defects, but there is some definite evidence that patients with lesions of the SLF and AF had symptoms of conduction aphasia subtypes (Axer et al., 2001).

Growing language-related connectivity studies of neuronal fiber bundles have employed diverse diffusion-weighted magnetic resonance imaging (dMRI; Le Bihan and Breton, 1985) methods, which allowed the mapping of macrostructural connectivities in the human brain *via* the application of tractography or fiber tracking algorithms. Correspondingly, the language-related network of white matter connections, such as the frontoparietal and frontotemporal white matter tracts, has been demonstrated by various structural connectivity studies *in vivo* and compared with many functional imaging studies (Duffau et al., 2002, 2005; Powell et al., 2006; Duffau, 2008; Hua et al., 2009; Saur et al., 2010).

However, the exact anatomical location and termination of the dorsal language pathway remain under debate (Brauer et al., 2011; Dick and Tremblay, 2012). Although consensus about SLF and AF division has been formed to some extent more recently (Table 1), slight differences persist regarding the exact cortical locations where the fiber tracts originate or terminate. In particular, the AF has been more disputed because the aspect of the fiber pathway is significantly different between human and nonhuman primates. In this regard, a recent study (Tremblay and Dick, 2016) has reported that the current language model could not represent the distributed connectivity relevant to the language property due to the use of outdated brain anatomy, which means that various subregions have not been thoroughly considered. Therefore, it is crucial to define subcomponents of language-related connectivity using improved brain models and dissociate their functional roles considering clinical interaction to identify more exact language-related regions.

**Table 1:** Contemporary and sometimes contentious models of the superior longitudinal fasciculus (SLF) and arcuate fasciculus (AF) connectivity.

**Study groups**	**Fiber tracts**	**Interconnected regions**	
		**Originated regions**	**Terminated regions**
Catani et al. (2005)	SLF and AF	Direct temporo-frontal pathway and indirect temporal-parietal-frontal pathway	
Makris et al. (2005)	SLF-I	Superior and medial parietal cortex	Dorsal parts of the frontal region
	SLF-II	Posterior-inferior parietal region	Prefrontal region
	SLF-III	Supramarginal gyrus	Brodmann's area of 6, 44, and 46
	AF	Caudal superior temporal region	Brodmann's area of 8 and 46
Thiebaut et al. (2011)	SLF-I	Precuneus and the superior parietal lobe	Superior frontal and anterior cingulate gyri
	SLF-II	Anterior intermediate parietal sulcus and the angular gyrus	Posterior parts of the superior and middle frontal gyri
	SLF-III	Temporo-parietal junction	Inferior frontal gyrus
Kamali et al. (2014)	SLF-I	Superior and medial parietal cortex	Dorsal and medial cortex of the frontal lobe
	SLF-II	Posteriolateral parts of the parietal cortex	Dorsolateral prefrontal cortex
	SLF-III	Rostral portion of the inferior paretal lobe	Ventral premotor and prefrontal cortex
	AF	Superior temporal gyrus	Dorsolateral prefrontal cortex
Yagmurlu et al. (2016) and Wang et al. (2017)	SLF-I	Precuneus	Anterior cingulate cortex and posterior parts of the superior frontal gyrus
	SLF-II	Angular gyrus	Middle frontal gyrus
	SLF-III	Supramarginal gyrus	Pars opercularis
Glasser and Rilling (2008) and Yagmurlu et al. (2016)	Dorsal-AF	Posterior parts of the middle and inferior temporal gyrus	Ventral premotor cortex, pars opercularis, posterior middle frontal gyrus
	Ventral-AF	Middle and posterior parts of the superior temporal gyrus	Ventral premotor cortex, pars opercularis and triangularis

In this study, we scrutinized the structural connectivity of the frontoparietal and frontotemporal fiber tracts in the left hemisphere using magnetic resonance (MR) diffusion tractography and the track termination distribution on the finer subregions defined by the Brainnetome atlas (Jiang, 2013; Fan et al., 2014, 2016). This study focuses on understanding the connectivity distribution of the dorsal language pathways and searching for anatomically more elaborate language-related cortical regions, specifically in the prefrontal cortex (PFC), inferior parietal cortex (IPC), and temporal cortex (TC).

## Materials and Methods

### Dataset

In the Human Connectome Project (HCP) dataset (Van Essen et al., 2013), 1,200 subjects were categorized as “HCP young adult” containing structural, functional, and diffusion MRI data. Since the 7.0T dMRI has the highest spatial resolution in the HCP protocol, i.e., 1.05 mm isotropic voxels, 169 subjects whose dataset contained the 7.0T dMRI were finally selected. The subjects' ages ranged from 20 to 35 years, while the male-to-female ratio was 67:102. For diffusion-weighted echo-planar imaging (EPI), the imaging parameters of the HCP protocol were as follows: *voxel size* = 1.05 × 1.05 × 1.05 *mm*^3^; *frames* = 71; *TR*/*TE* = 7, 000/71.2 *ms*; *b*−*values* = 1, 000 and 2, 000 *s*/*mm*^2^; and *number of diffusion directions* = 143.

The dMRI data were provided as preprocessed by the HCP group according to the HCP diffusion pipeline performing susceptibility correction, eddy current and motion correction, gradient nonlinearities correction, and linear registration to native T1-weighted volume. For an in-depth description of the HCP protocol for data acquisition, processing, and analysis, refer to the 1,200 subjects release reference manual of the HCP (https://www.humanconnectome.org/storage/app/media/documentation/s1200/HCP_S1200_Release_Reference_Manual.pdf; Andersson et al., 2003; Glasser and Van Essen, 2011; Sotiropoulos et al., 2013; Van Essen et al., 2013).

### Series of Data Processing Steps

**Figure 2:**
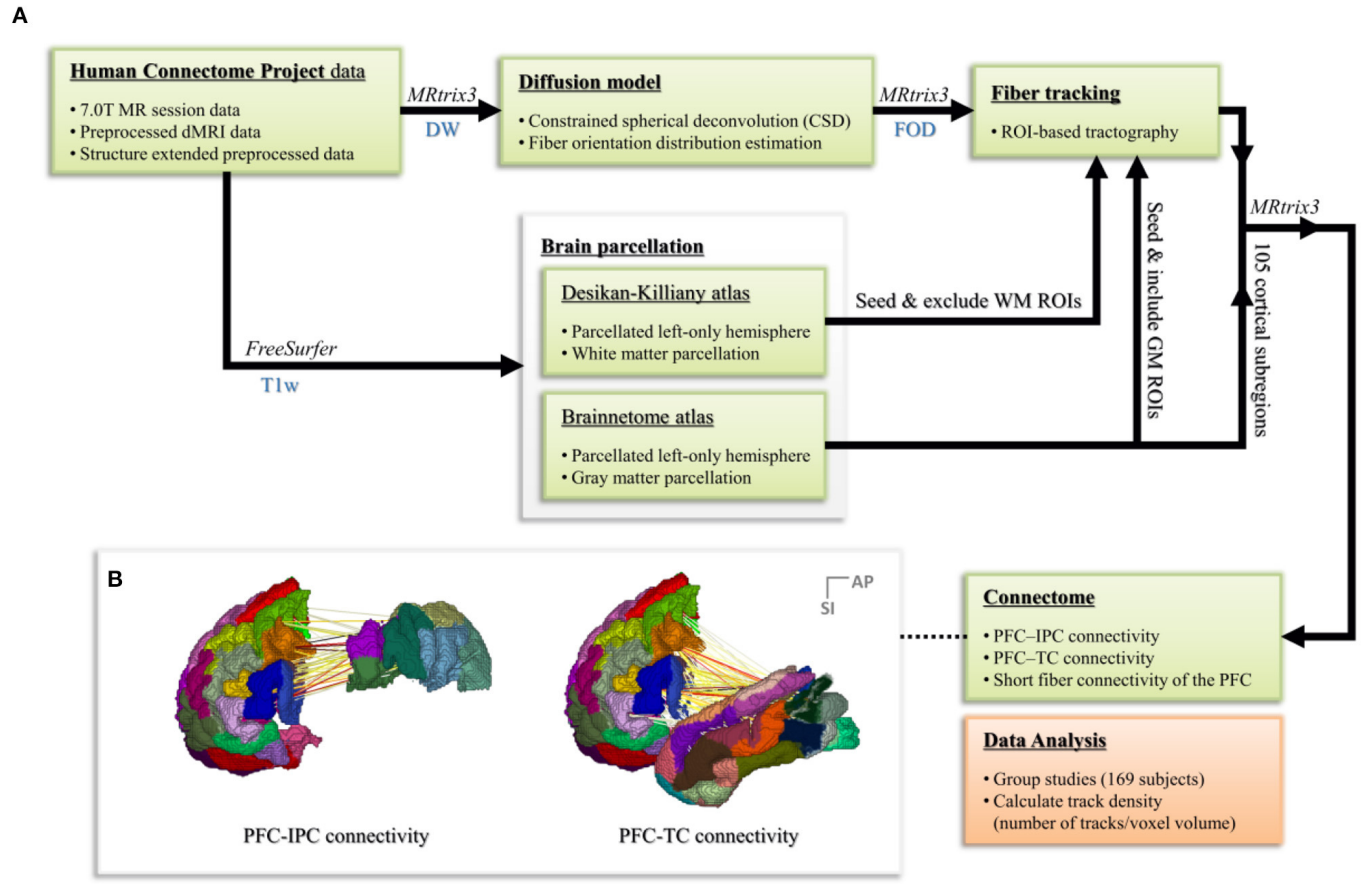
**(A)** The workflow of the whole data processing steps. **(B)** Connectome process based on the parcellated Brainnetome atlas node for regions of interest.

During the data processing steps, the fiber orientation distribution (FOD) estimation method using the constrained spherical deconvolution (CSD) algorithm was utilized, which requires the acquiring of data using the high angular resolution diffusion-weighted imaging strategy (HARDI; Frank, 2001; Tuch et al., 2002; Tournier et al., 2004, 2007). Tractography based on the FOD in each voxel and connectome process was performed using the MRtrix3 software package (J-D Tournier, Brain Research Institute, Melbourne, Australia; https://github.com/MRtrix3/mrtrix3; Tournier et al., 2012). To conduct a study that specifically observes cognitive and linguistic abilities, only the left hemisphere of the brain was considered. The workflow of the entire data processing step is shown in Figure 2.

#### Fiber Orientation Estimation

To estimate the full FOD function, CSD was performed on the MR signal data with a response function that describes a single coherently aligned white matter fiber population. This approach directly measures the signal orientation from the diffusion-weighted signal profile contained within each voxel. The obtained FOD contained orientation information and the corresponding volume fractions within each voxel (Tournier et al., 2004, 2007, 2008).

Recent studies have set the minimum value of the sampling density required for the proper characterization of diffusion-weighted data. The signal profile measured *in vivo* contains significant information up to the spherical harmonics of order 8 (Tournier et al., 2011, 2013). Therefore, in this study, each FOD was estimated from the diffusion-weighted data using the CSD algorithm with a lmax = 8.

#### Parcellation and Tractography

The preprocessed T1-weighted images, more specifically segmented images of gray matter and white matter, were parcellated using the Brainnetome (http://atlas.brainnetome.org; Jiang, 2013; Fan et al., 2014; Fan et al., 2016) and Desikan-Killiany (DK) atlas, respectively, using FreeSurfer software (http://surfer.nmr.mgh.harvard.edu/; Fischi, 2012). The Brainnetome atlas was used to parcellate the cortical regions of interest (ROI), while the DK atlas was additionally used for white matter parcellation since the Brainnetome atlas provides only cortical parcellation information. Gray matter parcellation labels for the Brainnetome atlas were mapped to each subject using anatomical surface data. In contrast, the white matter parcellation volume based on the DK atlas was generated in the segmentation steps by FreeSurfer.

This study mainly concentrated on the PFC, IPC, and TC regions. PFC was defined as Brodmann's area (BA) 8–14 and 44–47, which is located in the frontal portion of the frontal cortex (Murray and Wise, 2017). IPC was defined as BA 39 and 40, while TC was defined as BA 20–22, 28, 34–38, and 41–42. Based on the Brainnetome atlas, PFC, IPC, and TC were further divided into 23, 6, and 28 subregions, respectively.

To find the dorsal structural connectivity, such as the SLF and AF, the PFC, IPC, and TC were used as ROI for the tractography. Therefore, SLF (SLF-II and SLF-III) was set by starting from the IPC and ending in the PFC, and AF was set by starting from the TC and ending in the PFC. Finally, short fiber connectivity was generated to analyze the connectivity within the PFC.

**Figure 3:**
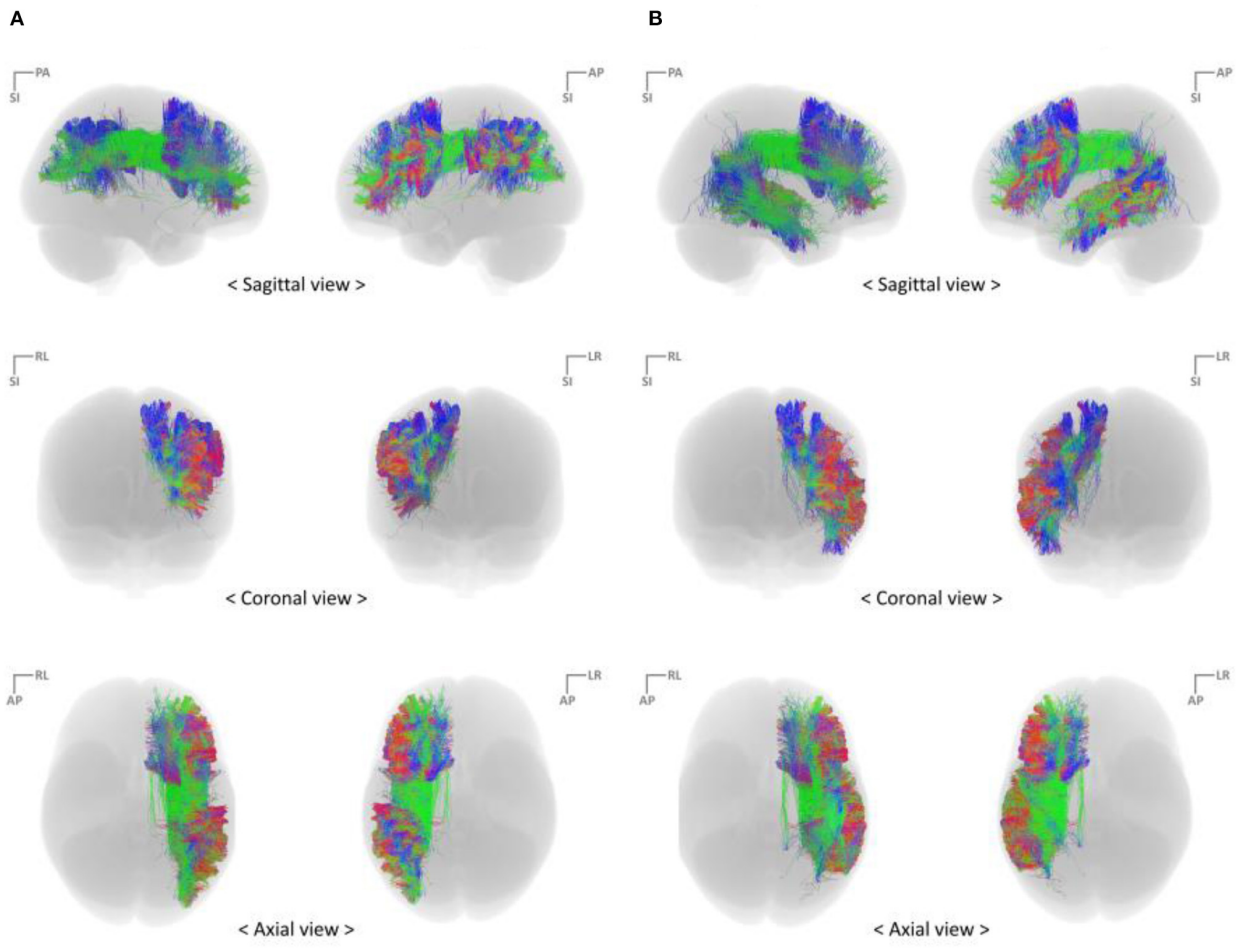
Reconstruct fiber bundles using the high angular resolution diffusion-weighted imaging strategy **(A)** PFC–IPC fiber tracks **(B)** PFC–TC fiber tracts. IPC, inferior parietal cortex; PFC, prefrontal cortex; TC, temporal cortex.

With the parcellated gray matter and white matter information, probabilistic tractography was performed using the iFOD2 algorithm implemented in MRtrix3 to investigate the structural connectivity between PFC and IPC and between PFC and TC (Table 2, Figure 3). Relevant tracking parameters were track minimum/maximum length = 60/180 mm; algorithm step size = 0.5 mm; curvature radius constraint = 0.8 mm; and FOD cutoff for track termination = 0.05. Streamline seeding was performed on the white matter because the diffusion-weighted signal at the gray matter is relatively small, which could result in track distortion at the white matter–gray matter interface. Furthermore, the end points of all the streamlines that cross the white matter–gray matter interface were precisely cropped, and anatomically constrained tractography was used to produce anatomically plausible streamlines that originate and terminate in the gray matter (Smith et al., 2012). The adequate values of step size and curvature constraint were determined through a parameter optimization process streamlined to the number of seeds. Additionally, to conduct a study that observes only linguistic abilities and their associated cognitive abilities, the right hemisphere of the brain was excluded, and irrelevant or unwanted cortexes were excluded considering the cortico-cortical connection property of the association fiber tracts.

**Table 2:** Guideline for reconstructing main fiber tracts.

**Fiber tract**	**Streamline seeding region (white matter)**	**Included region (gray matter)**	**Excluded regions (gray matter)**	**Number of tracks per subject**	**Reference figures**
PFC-IPC connectivity (SLF-II and SLF-III)	Inferior parietal gyrus	Prefrontal cortex	Temporal cortex, insular cortex, limbic cortex, occipital cortex, and subcortex	10,000	Figure 3A
PFC-TC connectivity (AF)	Temporal gyrus	Prefrontal cortex	Parietal cortex, insular cortex, limbic cortex, occipital cortex, and subcortex	10,000	Figure 3B
Short fiber connectivity within the PFC	Frontal lobe	Prefrontal cortex	Temporal cortex, parietal cortex, insular cortex, limbic cortex, occipital cortex, and subcortex	10,000	Figure 3C

#### Connectome

The connectivity matrix per subject was calculated based on the generated tractography streamlines and a node parcellation image that considered the ROI of this study using the connectome algorithm implemented in MRtrix3 (Smith et al., 2015; Tournier et al., 2019). In this process, we set the option to perform a radial search from the end point of each streamline to locate the nearest node within 2 mm.

The parcellated T1-weighted image was used to estimate the correlation matrix or track density matrix for the structural connectivity between subregions. These track density matrices were obtained by including the end points of each fiber on the cortical gray matter that matched the Brainnetome areas. Each track's density was calculated as the number of tracks that reached each subregion per voxel volume ratio of seeding subregions, which were randomly seeded and streamlined within a seed mask image. In other words, the calculated track density represents the connectivity ratio of tracks that reached the seeding subregions, which was set based only on the volume of the randomly seeded streamlines.


(1) $$\begin{array}{l} \text{Track density}\\ =\frac{\text{Number of reached tracks for each subregion}}{\frac{\text{Seeding subregion volume}}{\text{Seeding total volume}}\times \text{Number of total streamlines}} \end{array}$$

### Data Analysis

#### Finding Major Subregions Connected *via* Fiber Tracts

The average track density matrix indicates the mean value of the calculated track density between the set subregions of 169 subjects. Based on this average track density matrix, the PFC subregions in which the number of reached tracks is in the 95th percentile were considered major subregions that have significant connectivity with the seeding areas. The number of tracks reaching the major PFC subregions was analyzed using box-and-whisker plots showing the distribution for the 169 subjects.

#### Three-Dimensional (3D) Visualization of Structural Connectivity

The average track density matrix was also visualized as a 3D brain. In the 3D visualization, each cortical subregion indicated the sum of the average track density related to that subregion and was represented by the relative density of a certain color across all ROI.

#### Mapping End Points of Streamlines to the IPC and TC

The opposite end points of the streamlines reaching the major PFC subregion were mapped to the seeding regions to confirm the connectivity distribution and find more specific anatomical connectivity locations within the seeding regions. For this process, the other fiber tracts, which were set to originate from each major PFC subregion, were generated. Relevant tracking parameters were set the same as the PFC-IPC and PFC-TC connectivities (Table 3).

**Table 3:** Guideline for reconstructing fiber tracts originated from the major PFC subregions.

**Fiber tract**	**Streamline seeding region (gray matter)**	**Included region (gray matter)**	**Excluded regions (gray matter)**	**Number of tracks per subject**
IFJ-IPC connectivity	Inferior frontal junction	Inferior parietal cortex	Temporal cortex, insular cortex, limbic cortex, occipital cortex, and subcortex	10,000
A44v-IPC connectivity	A44v (ventral area of BA44)	Inferior parietal cortex	Temporal cortex, insular cortex, limbic cortex, occipital cortex, and subcortex	10,000
IFJ-TC connectivity	Inferior frontal junction	Temporal cortex	Temporal cortex, parietal cortex, insular cortex, limbic cortex, occipital cortex, and subcortex	10,000
A44v-TC connectivity	A44v (ventral area of BA44)	Temporal cortex	Temporal cortex, parietal cortex, insular cortex, limbic cortex, occipital cortex, and subcortex	10,000

Fiber tracts for each subject were registered to Montreal Neurological Institute (MNI) space using a warping image, which was estimated by the HCP group from native T1-weighted structural space to MNI152 space *via* linear and nonlinear registration. The end points of the transformed fiber tracts to the reached cortical subregions were visualized by track density maps. The track mapping algorithms utilized in this process were implemented in MRtrix3 (Calamante et al., 2010).

## Results

### Language-Related Connectivity in the Human Brain

#### Connectivity Between PFC and IPC

**Figure 4:**
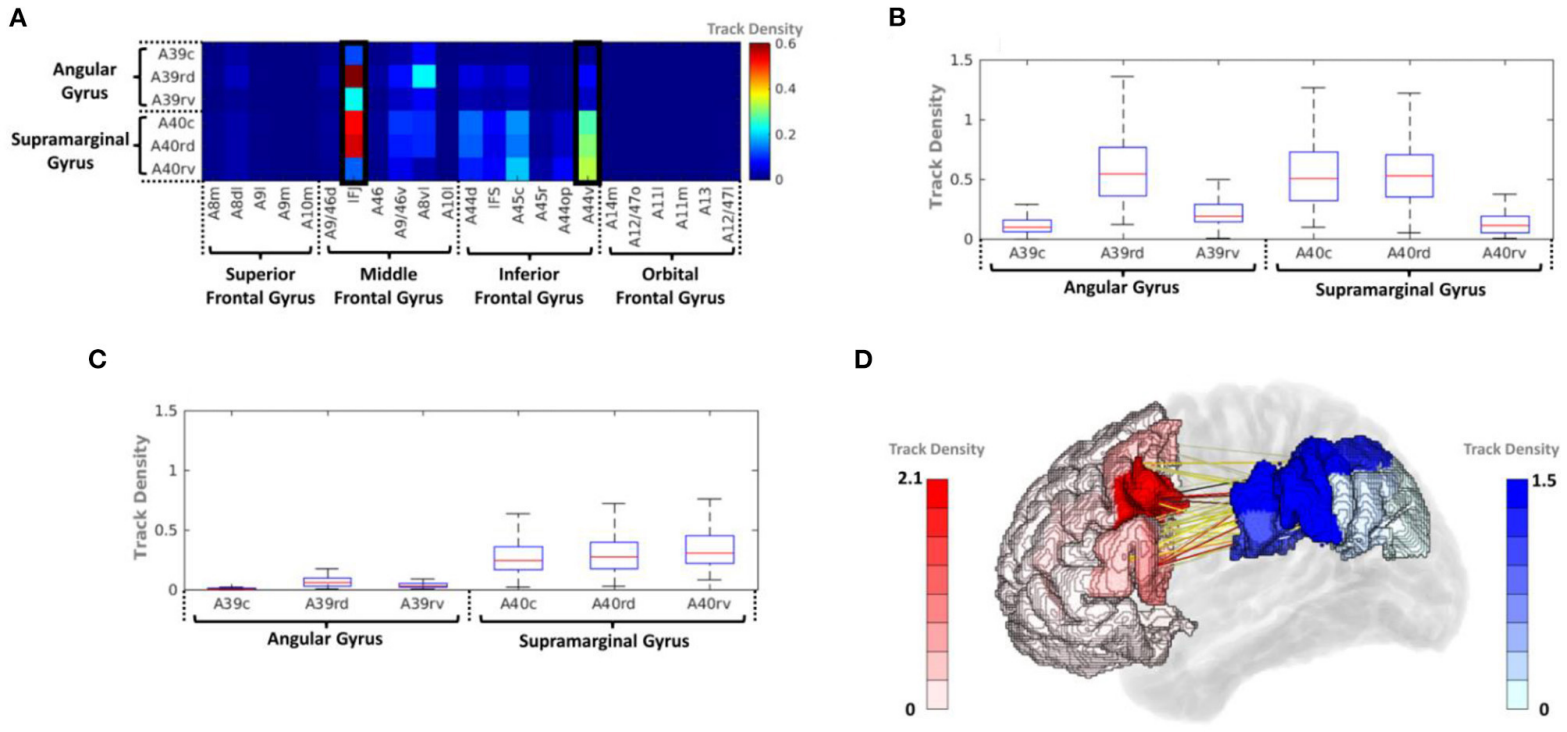
**(A)** Average track-density matrix of the acquired fiber tracts from the PFC–IPC. **(B)** Box-and-whisker plot showing the IFJ–IPC connectivity specifically for the regions indicated in **(A)**. **(C)** Box-and-whisker plot showing the A44v connectivity specifically for the regions indicated in **(A)**. **(D)** Three-dimensional visualization of track density in PFC–IPC connectivity. A8m, medial area 8; A8dl, dorsolateral area 8; A8vl, ventrolateral area 8; A9l, lateral area 9; A9m, medial area 9; A10m, medial area 10; A9/46d, dorsal area 9/46; A10l, lateral area 10; A11l, lateral area 11; A11m, medial area 11; A12/47l, lateral area 12/47; A12/47o, opercular area 12/47; A13, area 13; A14m, medial area 14; A39c, caudal area 39; A39rd, rostrodorsal area 39; A39rv, rostroventral area 39; A40c, caudal area 40; A40rd, rostrodorsal area 40; A40rv, rostroventral area 40; A44d, dorsal area 44; A44op, opercular area 44; A44v, ventral area 44; A45c, caudal area 45; A45r, rostral area 45; A46, area 46; IFJ, inferior frontal junction; IFS, inferior frontal sulcus.

The average track density matrix for the structural connectivity between the PFC and IPC is shown in Figure 4A. The matrix shows the mean value of the track density for all subjects. Among the PFC subregions, the inferior frontal junction (IFJ) and ventral area of BA44 (A44v) were considered the major PFC subregion having the connectivity to the IPC (Supplementary Table 1). Figures 4B,C show the corresponding box-and-whisker plots of IFJ and A44v, respectively, connected to subregions of the IPC for all subjects. As the result, the IFJ had the highest average track density with A39rd (rostrodorsal area of BA39), A40rd (rostrodorsal area of BA40), and A40c (caudal area of BA40). The A44v had relatively strong connectivity with A40rv (rostroventral area of BA40), A40rd, and A40c. The connectivity of the PFC subregions with the whole IPC (red) and the IPC subregions with the whole PFC (blue) was demonstrated in Figure 4D. It was clearly seen that most fiber tracts were connected to the IFJ/A44v and the anterior parts of the IPC with the IPC and the PFC, respectively (Supplementary Figure 1).

#### Connectivity Between PFC and TC

**Figure 5:**
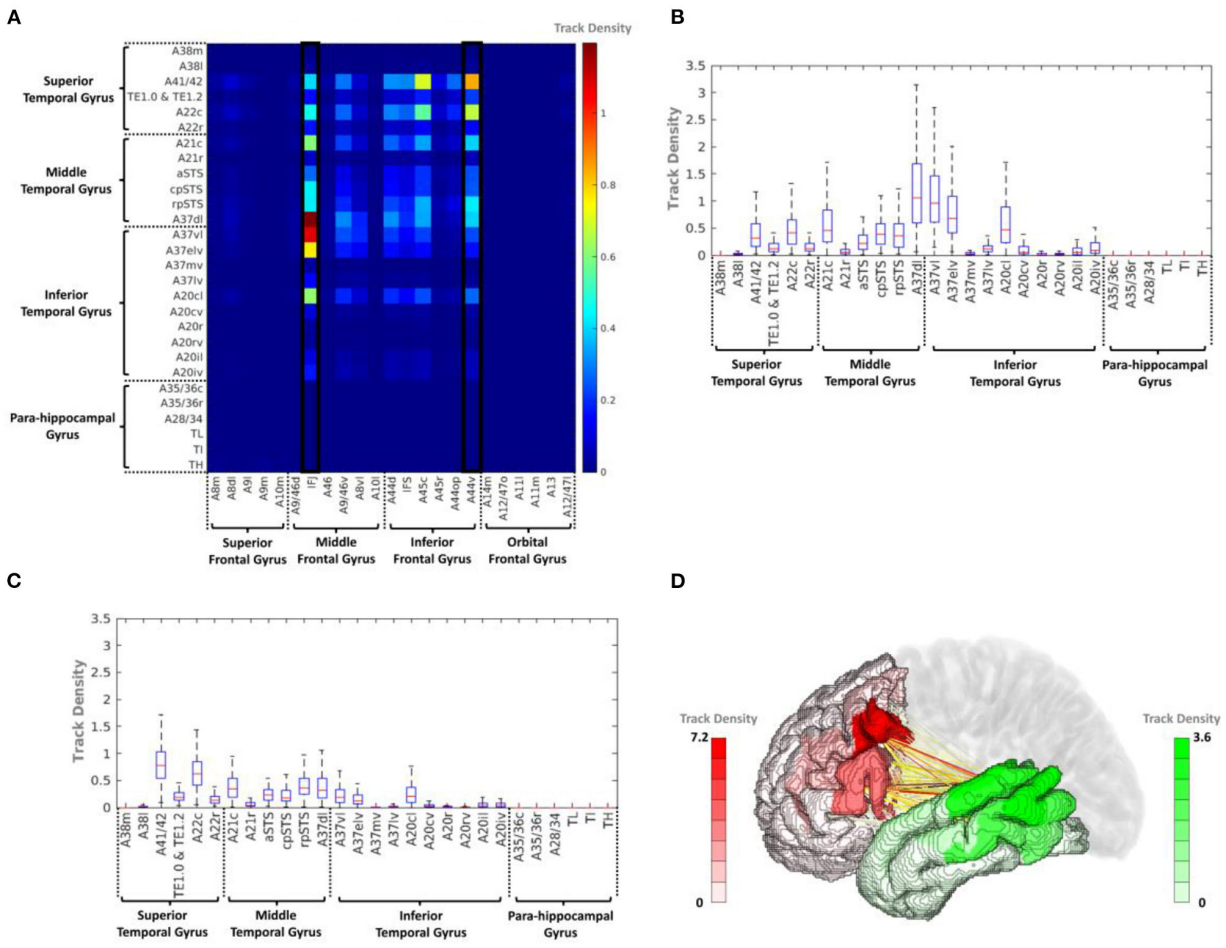
**(A)** Average track-density matrix of the acquired fiber tracts from the PFC–TC. **(B)** Box-and-whisker plot showing the IFJ–TC connectivity specifically for the regions indicated in **(A)**. **(C)** Box-and-whisker plot showing the A44v–TC connectivity specifically for the regions indicated in **(A)**. **(D)** Three-dimensional visualization of track density in PFC–TC connectivity. A8m, medial area 8; A8dl, dorsolateral area 8; A8vl, ventrolateral area 8; A9l, lateral area 9; A9m, medial area 9; A9/46d, dorsal area 9/46; A10m, medial area 10; A10l, lateral area 10; A11l, lateral area 11; A11m, medial area 11; A12/47l, lateral area 12/47; A12/47o, opercular area 12/47; A13, area 13; A14m, medial area 14; A21c, caudal area 21; A21r, rostral area 21; A22c, caudal area 22; A22r, rostral area 22; A37dl, dorsolateral area 37; A38l, lateral area 38; A38m, medial area 38; A41/42, area 41/42; A44d, dorsal area 44; A44op, opercular area 44; A44v, ventral area 44; A45c, caudal area 45; A45r, rostral area 45; A46, area 46; aSTS, anterior superior temporal sulcus; IFJ, inferior frontal junction; IFS, inferior frontal sulcus; TE, TE 1.0 and TE 1.2.

The average track density matrix between the PFC and TC is displayed in Figure 5A, which indicates the mean track density for all subjects. Similar to the PFC–IPC connectivity, IFJ and A44v were considered the major PFC subregions having the connectivity to the TC (Supplementary Table 2), and Figures 5B,C show the box-and-whisker plot showing the distribution of IFJ–TC connectivity and A44v–TC connectivity for the 169 subjects, respectively. As the result, the IFJ had the highest average track density with A37dl (dorsolateral area of BA37), A37vl (ventrolateral area of BA37), and A37elv (extreme lateroventral area of BA37). The A44v had relatively high connectivity with A41/42 (BA41 and 42) and A22c (caudal area of BA22). Furthermore, the A45c (caudal area of BA45) within the PFC also had a considerable average track density with A41/42 within the TC. The connectivity of the PFC subregions with the whole TC (red) and the TC subregions with the whole PFC (green) is visualized in Figure 5D. It was clearly shown that the most fiber tracts were connected to the IFJ/A44v and the superior and posterior parts of the TC with the TC and the PFC, respectively (Supplementary Figure 2).

### Fiber Tract Termination Distribution on the IPC and TC

#### Mapping IPC Streamline End Points

**Figure 6:**
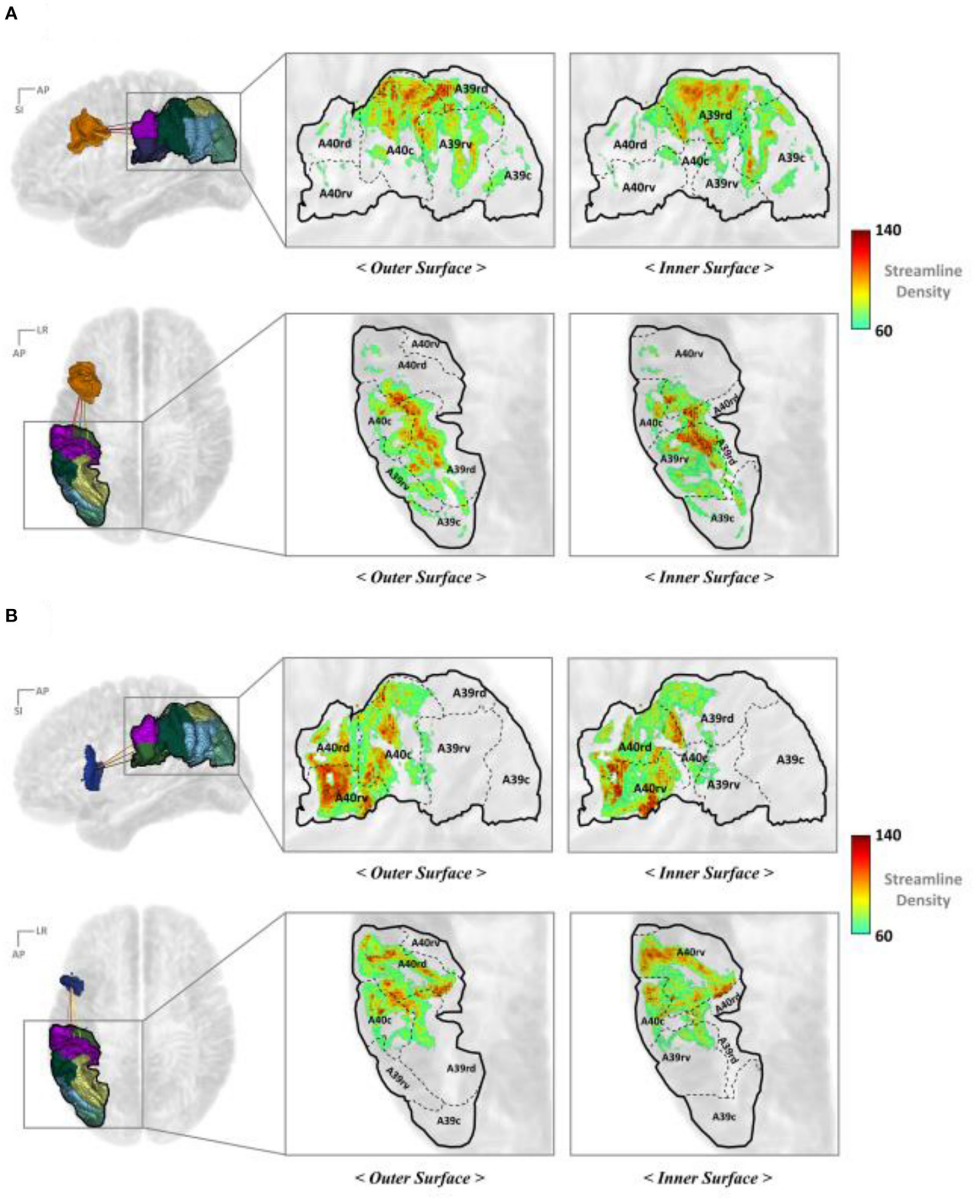
Mapping the end points of the streamline to the IPC **(A)** IFJ–IPC streamlines and **(B)** A44v–IPC streamlines. A8m, medial area 8; A8dl, dorsolateral area 8; A8vl, ventrolateral area 8; A9l, lateral area 9; A9m, medial area 9; A9/46d, dorsal area 9/46; A10l, lateral area 10; A10m, medial area 10; A11l, lateral area 11; A11m, medial area 11; A12/47l, lateral area 12/47; A12/47o, opercular area 12/47; A13, area 13; A14m, medial area 14; A39c, caudal area 39; A39rd, rostrodorsal area 39; A39rv, rostroventral area 39; A40c, caudal area 40; A40rd, rostrodorsal area 40; A40rv, rostroventral area; 40A46, area 46; A44d, dorsal area 44; A44op, opercular area 44; A44v, ventral area 44; A45c, caudal area 45; A45r, rostral area 45; IFJ, inferior frontal junction; IFS, inferior frontal sulcus.

Figures 6A,B illustrate the IPC map for the streamline end points that originated from the IFJ and A44v, respectively. These show a more specific anatomical location and topological analysis of the structural connectivity. The end point density of the streamlines originating from each major PFC subregion differed. The IFJ had strong connectivity with the superior parts of the IPC (A40c, A40rd, and A39rd), while the A44v, otherwise known as Broca's area, had strong connectivity with the anterior part of the supramarginal gyrus (A40rv, A40rd, and A40c). This allowed us to obtain more accurate cortical regions for each major fiber connectivity. Accordingly, this could be a further result of previous studies of SLF-II and SLF-III.

#### Mapping TC Streamline End Points

**Figure 7:**
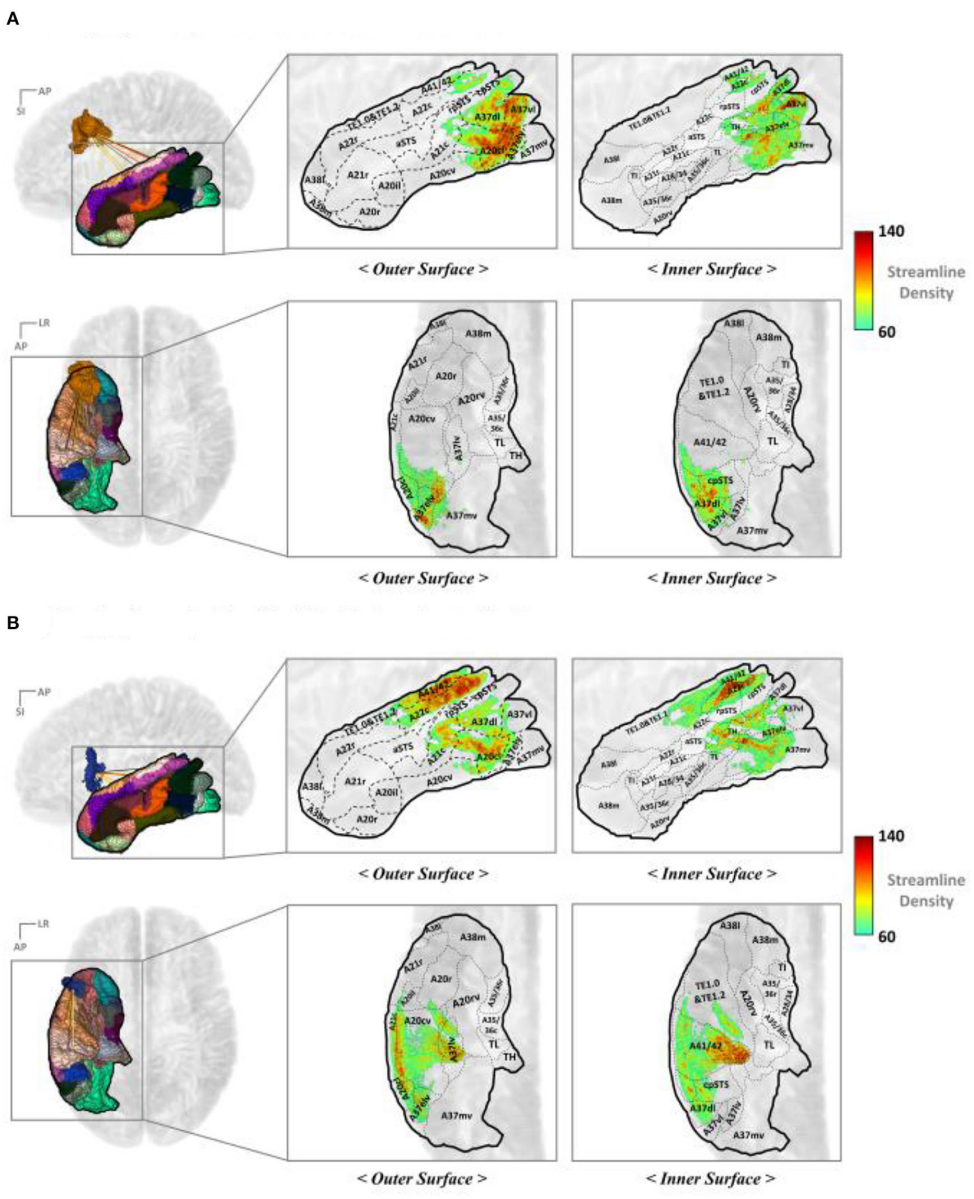
Mapping the end points of the streamlines to the TC: **(A)** IFJ–TC streamlines and **(B)** A44v–TC streamlines. A8dl, dorsolateral area 8; A8m, medial area 8; A8vl, ventrolateral area 8; A9l, lateral area 9; A9m, medial area 9; A9/46d, dorsal area 9/46; A10l, lateral area 10; A10m, medial area 10; A11l, lateral area 11; A11m, medial area 11; A12/47l, lateral area 12/47; A12/47o, opercular area 12/47; A14m, medial area 14; A13, area 13; A21c, caudal area 21; A21r, rostral area 21; A22c, caudal area 22; A22r, rostral area 22; A37dl, dorsolateral area 37; A38l, lateral area 38; A38m, medial area 38; A41/42, area 41/42; A44d, dorsal area 44; A44op, opercular area 44; A44v, ventral area 44; A45c, caudal area 45; A45r, rostral area 45; A46, area 46; aSTS, anterior superior temporal sulcus; IFJ, inferior frontal junction; IFS, inferior frontal sulcus; TE, TE 1.0 and TE 1.2.

Figure 7 represents the TC mapping of the end points of the streamlines that connect the TC with the IFJ or A44v. In the case of TC, the cortical subregions linked with IFJ and A44v were separated more clearly. In summary, BA37, also known as the fusiform gyrus, has significant connectivity with the IFJ, while the A44v (part of Broca's area) shows strong connectivity with A41/42, which is known as the auditory cortex, and A22c (otherwise known as Wernicke's area). This finding is consistent with previous study results of the AF being divided into dorsal and ventral portions and provides more specific anatomical locations.

### Short Fiber Connectivity Within the PFC

Overall, the connectivity results indicated that IFJ and A44v had the strongest structural connectivity in the SLF and AF fiber bundles, indicating that these subregions could play a vital role in language tasks. Therefore, we assume that IFJ and A44v also have considerable connectivity with language-related regions within the PFC.

**Figure 8:**
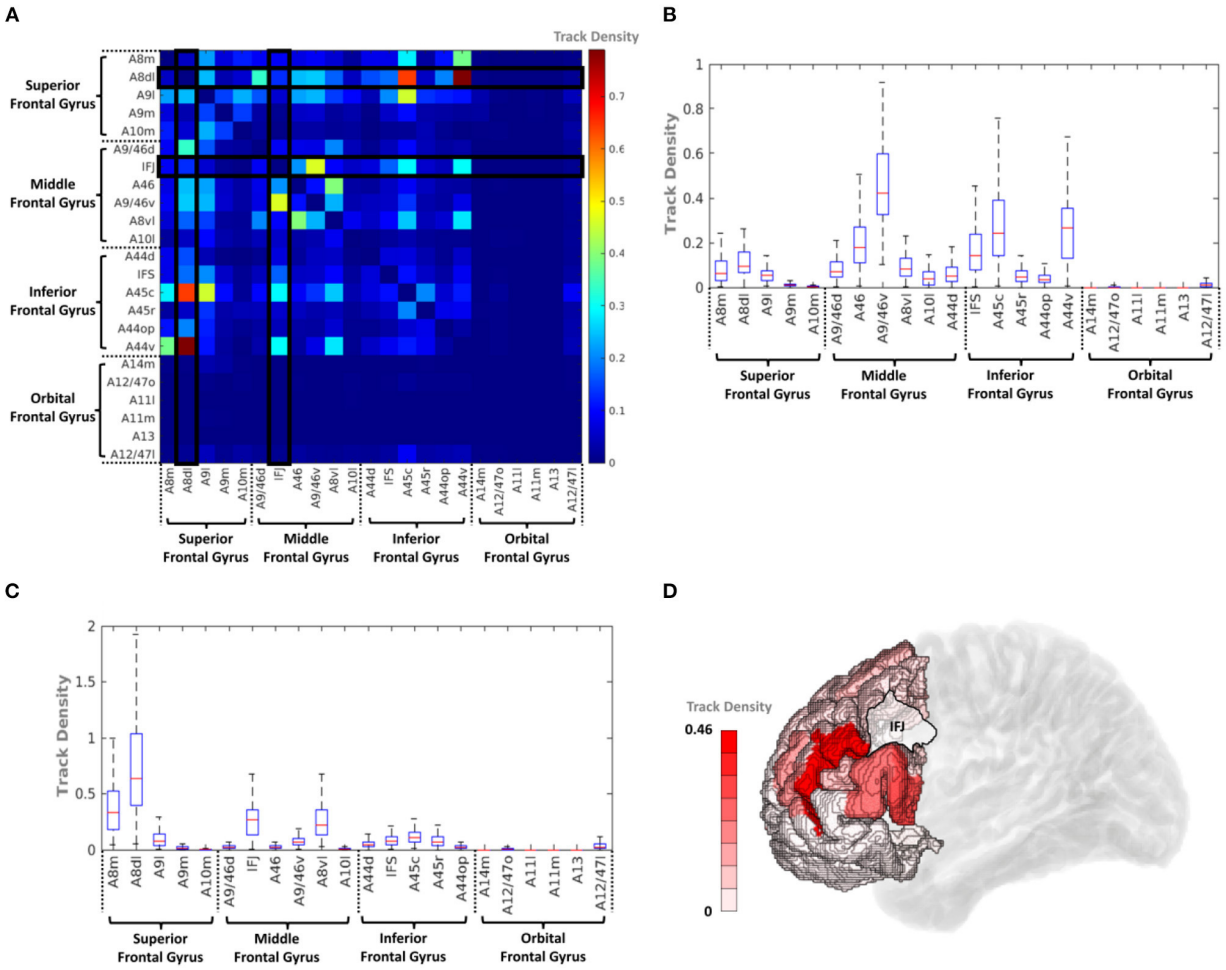
**(A)** Average track density matrix of the acquired short fiber tracts from the PFC. **(B)** Box-and-whisker plot showing the PFC–IFJ connectivity specifically for the regions indicated in **(A)**. **(C)** Box-and-whisker plot showing the PFC–A44v connectivity specifically for the regions indicated in **(A)**. **(D)** Three-dimensional visualization of track density within PFC–IFJ focused short fiber connectivity. A8dl, dorsolateral area 8; A8m, medial area 8; A9l, lateral area 9; A9m, medial area 9; A9/46d, dorsal area 9/46; A10m, medial area 10; A8vl, ventrolateral area 8; A10l, lateral area 10; A11l, lateral area 11; A11m, medial area 11; A12/47l, lateral area 12/47; A12/47o, opercular area 12/47; A13, area 13; A14m, medial area 14; A44d, dorsal area 44; A45c, caudal area 45; A45r, rostral area 45; A44op, opercular area 44; A44v, ventral area 44; A46, area 46; IFJ, inferior frontal junction; IFS, inferior frontal sulcus.

To analyze the short fiber connectivity within the PFC, the average track density matrix was produced by applying the same tractography process to the PFC area (Figure 8A). Figures 8B,C show corresponding box-and-whisker plots of the distribution of the IFJ and A44v connectivities indicated in the average track density matrix. The resulting short fiber connectivity showed that A44v had the highest mean value with A8dl (dorsolateral area of BA8), and the IFJ had a relatively high average track density with A9/46v (ventral area of BA9 and BA46). Considering the IFJ had the highest connectivity with both IPC and TC, IFJ-focused short fiber connectivity was visualized in Figure 8D. It was distinctly shown that most fiber tracts from the IFJ were interconnected with A9/46v (Supplementary Figure 3).

## Discussion

**Figure 9:**
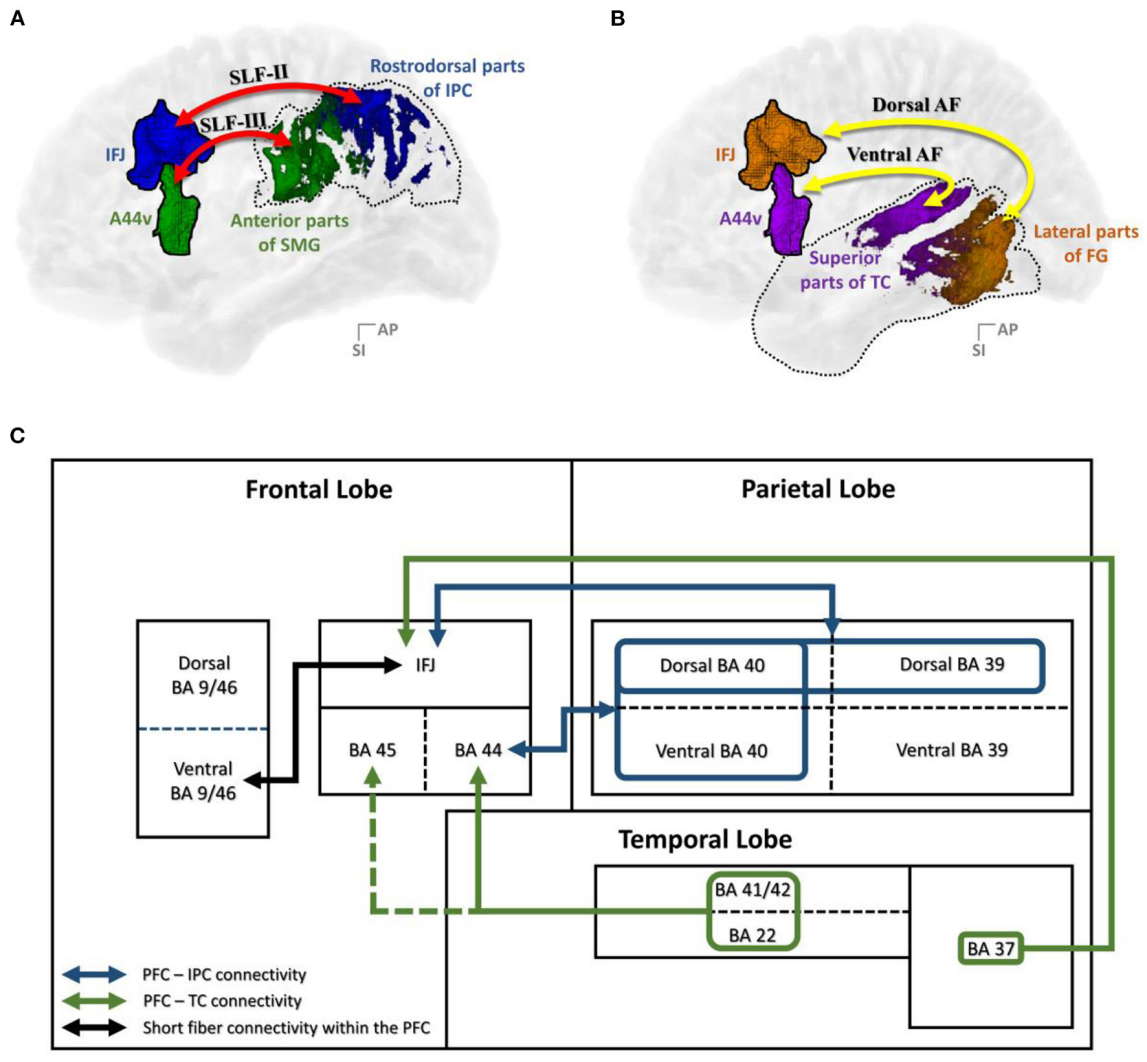
Summary of the results: **(A)** PFC–IPC connectivity; **(B)** PFC–TC connectivity; **(C)** A block diagram of the structural connectivity of the dorsal language pathway. A44v, ventral area of BA44; AF, arcuate fasciculus; AG, angular gyrus; DLPFC, dorsolateral prefrontal cortex; FG, fusiform gyrus; IFJ, inferior frontal junction; IPC, inferior parietal cortex; PFC, prefrontal cortex; SLF, superior longitudinal fasciculus; SMG, supramarginal gyrus; TC, temporal cortex.

In this study, brain regions that were interconnected *via* the SLF and AF were examined and further specified, along with various previous studies. As shown in Figures 9A,B, the structural connectivity that links the PFC with the IPC (i.e., the SLF-II and SLF-III) and the TC (i.e., the AF) showed that the IFJ and A44v of the PFC had significant connectivity. Furthermore, the rostrodorsal region of the IPC and the lateral part of the fusiform gyrus had the strongest connection to the IFJ, while the anterior part of the supramarginal gyrus and the superior regions of the TC had significant connectivity with the A44v.

To be more concrete, we specified the middle frontal gyrus as the IFJ, which had the major white matter connectivity with parts of the SLF-III and the dorsal AF. Among the previous connectivity studies of the language pathway, there is consensus that the SLF-III and the dorsal AF are connected to the middle frontal gyrus (Makris et al., 2005; Glasser and Rilling, 2008; Thiebaut et al., 2011; Kamali et al., 2014; Wang et al., 2016; Yagmurlu et al., 2016). Based on our results, we confirmed that IFJ within the middle frontal gyrus had significant connectivity with the IPC and the TC. A previous cognitive study identified that the IFJ area lies at the junction of the premotor and the PFC as part of the mid-dorsolateral PFC (DLPFC), which is located at the junction of the premotor, language, and working memory domains (Brass et al., 2005). Moreover, there has been much speculation that the IFJ is ideally located to promote the interaction of information among these domains based on various functional imaging studies (Derrfuss et al., 2004, 2005; Brass et al., 2005; Neumann et al., 2005). In this respect, the IFJ within the PFC might play a crucial role in language processing by communicating with the IPC and the TC.

In contrast, the inferior frontal gyrus, which was interconnected with the IPC and the TC *via* the SLF-III and the ventral AF, was further concretized as the ventral part of the pars opercularis (A44v). According to the previous studies, the SLF-III and the ventral AF are connected to the inferior frontal gyrus (Makris et al., 2005; Glasser and Rilling, 2008; Thiebaut et al., 2011; Kamali et al., 2014; Wang et al., 2016; Yagmurlu et al., 2016). Based on our results, the inferior frontal gyrus had strong structural connectivity; however, the ventral area of BA44 had much more direct connectivity with the IPC and the TC than other regions related to the inferior frontal gyrus. It is well-known that the pars opercularis (BA44) and pars triangularis (BA45) are associated with Broca's area and phonological rehearsal (Smith et al., 1998; Na et al., 2000; Baldo and Dronkers, 2006). Although there has been no clear functional separation between BA44 and BA45, A44v is expected to play an important role in the language circuitry.

Based on these major PFC subregions in our study, we investigated a more specific cortical region of IPC in which each fiber tract branch was interconnected. The superior portion (A39rd, A40c, and A40rd) and anterior parts (A40rv, A40rd, and A40c) of the IPC had the strongest connectivity with the IFJ and A44v, respectively. In this context, SLF-II and SLF-III are known to originate from the angular gyrus and the supramarginal gyrus, respectively (Makris et al., 2005; Thiebaut et al., 2011; Kamali et al., 2014; Wang et al., 2016; Yagmurlu et al., 2016). First, the superior portion of the IPC (A39rd, A40c, and A40rd) had the strongest connectivity with the IFJ. This connectivity could be considered the SLF-II; however, it slightly differed from previous studies in which it originated from the superior parts of the whole IPC rather than from the angular gyrus only. A previous subregional study (Ding et al., 2020) reported that damage to the rostrodorsal regions of the IPL resulted in impaired lexical selection and reduced structural complexity for speech production.

In contrast, the anterior parts of the IPC (A40rv, A40rd, and A40c) had significant connectivity with A44v *via* the SLF-III. This connectivity finding was consistent with those of previous studies in that it originated from the supramarginal gyrus (Makris et al., 2005; Thiebaut et al., 2011; Kamali et al., 2014; Wang et al., 2016; Yagmurlu et al., 2016). Furthermore, we identified that it was intensively interconnected with the anterior parts of the supramarginal gyrus. According to the dissociation analysis, the rostral supramarginal gyrus was significantly correlated with syntactic accuracy deficits along with the pars opercularis (Ding et al., 2020). Regarding these results, we believe that the SLF-II and SLF-III, which could be dissociated by specific originated regions, reflected different functions for grammatical word production and syntactic accuracy.

Similar to the SLF, more specific regions of the TC, in which each AF branch was interconnected, were investigated based on the major PFC subregions. For the ventral AF, the posterior superior temporal gyri (A41/42 and A22c), well-known interconnected regions *via* the classical AF pathway, showed strong direct connectivity with A44v. This result was consistent with that of the original AF study in which the AF originated from the superior temporal gyrus (Glasser and Rilling, 2008; Yagmurlu et al., 2016). Lesions in the posterior superior temporal gyrus could reportedly lead to abnormalities in phonological retrieval and speech articulation (Binder, 2015). Specifically, A22c, known as Wernicke's area, has been discussed as a region related to language comprehension abnormalities (Binder, 2015). Based on previous studies (Smith et al., 1998; Na et al., 2000; Baldo and Dronkers, 2006; Barbey et al., 2013; Binder, 2015) and the current results, the connectivity between A44v and the posterior superior temporal gyrus might play a crucial role in phonological pathways that convert the stimulus to a phonological form.

For the dorsal AF, the dorsolateral area of the fusiform gyrus (A37dl) within the TC showed the strongest connectivity with the IFJ. This AF branch originated from the posterior parts of the middle and inferior temporal gyrus (Glasser and Rilling, 2008; Yagmurlu et al., 2016). This result was in accordance with those of previous studies and provided further specific regions. The middle temporal gyrus, which includes the fusiform gyrus (BA37) region, was previously studied for its role in visual perception and lexical–semantic language function (Ardila et al., 2015; Fernández-Miranda et al., 2015; Jouen et al., 2015; Ding et al., 2020). In this regard, it was in line with the results of previous studies (Glasser and Rilling, 2008; Yagmurlu et al., 2016; Ding et al., 2020) that the dorsal AF could be related to the lexical–semantic language pathway.

Finally, realizing the importance of the IFJ and A44v, short fiber connectivity within the PFC was also observed. The resulting data showed that the IFJ has the strongest connectivity with A9/46v (otherwise known as the DLPFC; Figure 9C), which is known to activate the central executive system related to verbal working memory (Smith et al., 1998; Na et al., 2000; Baldo and Dronkers, 2006; Townsend et al., 2010; Barbey et al., 2013; Snow, 2016). Previous studies (Brass et al., 2005) proved the important relationship between working memory, language comprehension, and cognition. These studies provided evidence for the role of the frontoparietal and frontotemporal pathways in assisting language tasks that require working memory, such as a verbal working memory task or a repetition task that reproduces the comprehension of auditorily perceived words (Brass et al., 2005; Derrfuss et al., 2005). Therefore, we hypothesize that the IFJ may play a crucial role in supporting complex processing through some form of working memory-related mechanism as an entrance of the PFC into the language pathway.

This study definitely has the limitation of a small sample size. Although the 1,200 subjects were available in the HCP dataset, only 7.0T dMRI were selected within the whole dataset. In this study, the spatial resolution of the dMRI was important to discriminate the targeted fiber bundles from each other. In the given dMRI protocols of the HCP data, the 7.0T data was superior to the 3.0T data in spatial resolution. The voxel volume of 7.0T was ~0.6 times smaller than that of 3.0T. Although, using the high resolution of 7.0T dMRI, we could specify the cortical subregions connected to the language-related fiber tracts, the higher spatial resolution would be required to dissociate further specific terminate regions within the IFJ and A44v where each fiber tract (SLF and AF, respectively) reached.

This study utilized the structural information of tractography and the track density image based on dMRI to visualize the connected subregions. Our approach to the functional interpretation of the subregions relied only on the Brainnetome atlas and its previous functional findings. It can be improved with more concrete and certain interpretation if the dMRI is combined with the corresponding functional MRI study in further study.

Although the tractography using dMRI provides useful information for the structural connection of fiber bundles in the brain, it has inherent limitations that the estimated tracts differ from the real fibers within the white matter. Fiber tracts were probabilistically generated by the fiber tracking algorithm tracing streamlines based on the stepwise. Therefore, the track density data using the number of tractography streamlines could be problematic as a quantitative measure (Calamante, 2019), and it should be cautiously considered. To show the relative strength of the connectivity between subregions, track density was used as the qualitative or semiquantitative metric.

## Conclusion

This study demonstrated the subregional structure connectivity of the SLF and AF tracts in the context of the language pathway. The fiber track density within the subregions was calculated using MR diffusion tractography. Both fiber bundles had two main aspects. First, the rostrodorsal regions of the IPC and the lateral parts of the fusiform gyrus had the strongest connectivity with the IFJ within the PFC *via* the SLF-III and dorsal AF. Second, the anterior parts of the supramarginal gyrus and the superior region of the TC had significant connectivity with A44v within the PFC *via* the SLF-II and ventral AF, respectively. The IFJ also showed the strongest connectivity with the DLPFC among the rest of the PFC regions *via* short fiber tracts. In conclusion, this study emphasized specific language-related regions of the human brain to further understand language circuitry.

## Data Availability Statement

The original contributions presented in the study are included in the article/Supplementary Material, further inquiries can be directed to the corresponding author.

## Ethics Statement

The studies involving human participants were reviewed and approved by the Human Connectome Project, WU-Minn Consortium (Principal Investigators: David Van Essen and Kamil Ugurbil; 1U54MH091657) funded by the 16 NIH Institutes and Centers that support the NIH Blueprint for Neuroscience Research and by the McDonnell Center for Systems Neuroscience at Washington University. The patients/participants provided their written informed consent to participate in this study.

## Author Contributions

Y-EH and Y-DS contributed to the study design, Y-EH contributed in data analysis, interpretation, and manuscript writing. Y-DS directed the project. Y-DS and Y-BK interpreted the data and critically revised the manuscript. All authors gave their final approval and agreed to be accountable for all aspects of the work.

## Funding

This study was supported by the Brain Research Program of the National Research Foundation of Korea funded by the Ministry of Science and ICT (2017M3C7A1049026) and by the National Research Foundation of Korea (NRF) grant funded by the Korean government (MSIT) (no. NRF-2020R1A4A1019623).

## Conflict of Interest

The authors declare that the research was conducted in the absence of any commercial or financial relationships that could be construed as a potential conflict of interest.

## Publisher's Note

All claims expressed in this article are solely those of the authors and do not necessarily represent those of their affiliated organizations, or those of the publisher, the editors and the reviewers. Any product that may be evaluated in this article, or claim that may be made by its manufacturer, is not guaranteed or endorsed by the publisher.
