# Supplementary material for: Finding Cortical Subregions Regarding the Dorsal Language Pathway Based on the Structural Connectivity
Source: Front Hum Neurosci. 2022 May 2;16:784340. doi: 10.3389/fnhum.2022.784340 (PMC9108242; doi:10.3389/fnhum.2022.784340)
Supplement: Supplementary file 3 [file Data_Sheet_1.docx]

Supplementary Material

# Supplementary Figures and Tables


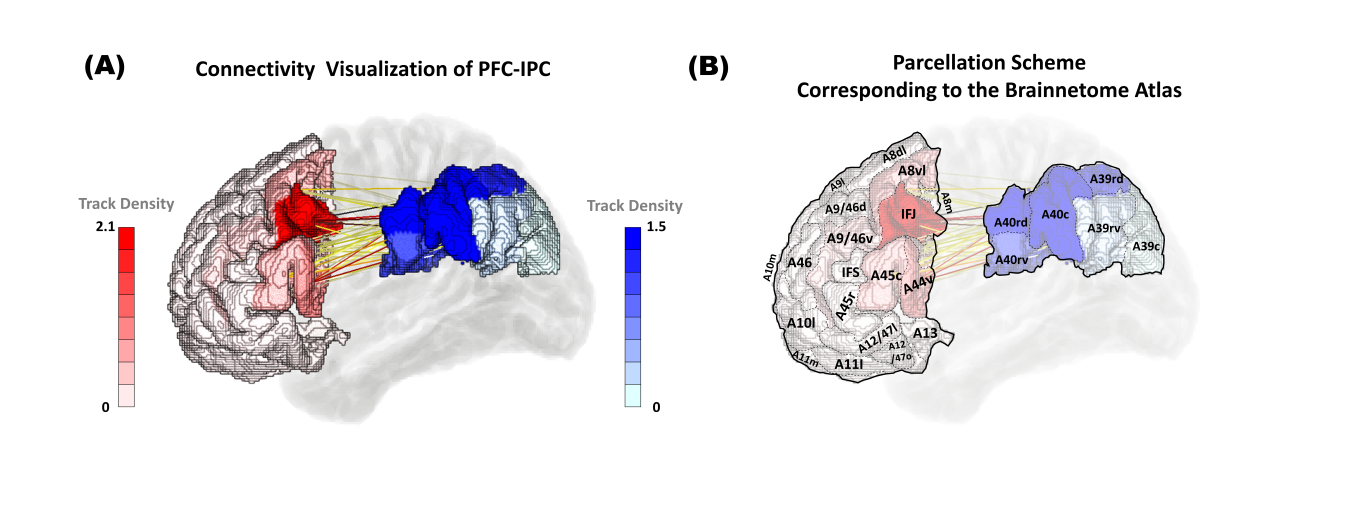


**Supplementary Figure 1.** **[A]** Connectivity visualization of PFC-IPC **[B]** Parcellation scheme corresponding to the Brainnetome atlas.

(**Abbreviation**: A8m(medial area 8), A8dl(dorsolateral area 8), A9l(lateral area 9), A9m(medial area 9), A10m(medial area 10), A9/46d(dorsal area 9/46), IFJ(inferior frontal junction), A46(area 46), A8vl(ventrolateral area 8), A10l(lateral area 10), A44d(dorsal area 44), IFS(inferior frontal sulcus), A45c(caudal area 45), A45r(rostral area 45), A44op(opercular area 44), A44v(ventral area 44), A14m(medial area 14), A12/47o(opercular area 12/47), A11l(lateral area 11), A11m(medial area 11), A13(area 13), A12/47l(lateral area 12/47), A39c(caudal area 39), A39rd(rostrodorsal area 39), A40rd(rostrodorsal area 40), A40c(caudal area 40), A39rv(rostroventral area 39), A40rv(rostroventral area 40))


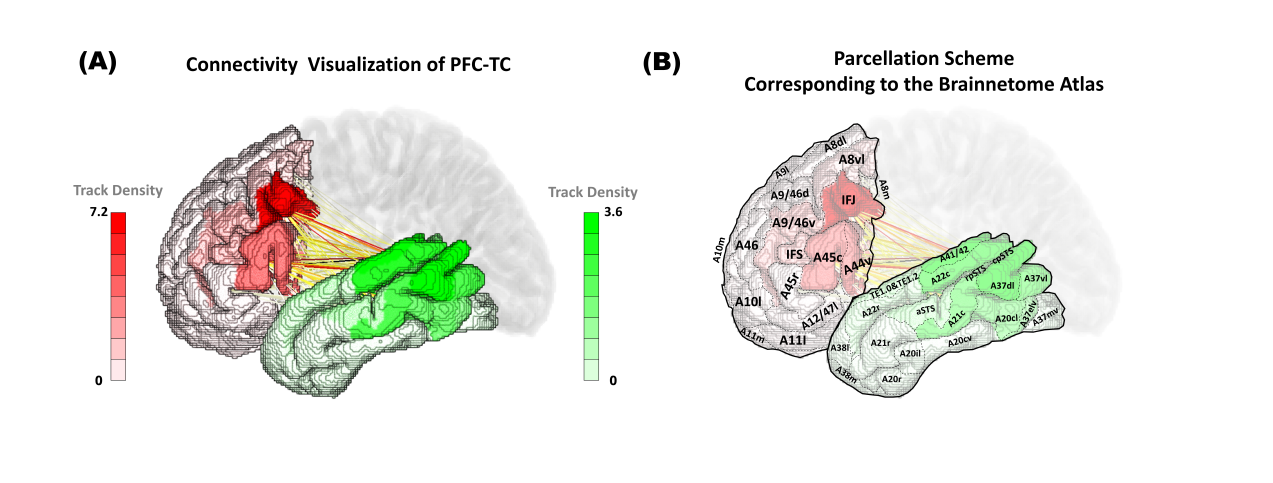


**Supplementary Figure 2. [A]** Connectivity visualization of PFC-TC **[B]** Parcellation scheme corresponding to the Brainnetome atlas.

(**Abbreviation**: A8m(medial area 8), A8dl(dorsolateral area 8), A9l(lateral area 9), A9m(medial area 9), A10m(medial area 10), A9/46d(dorsal area 9/46), IFJ(inferior frontal junction), A46(area 46), A8vl(ventrolateral area 8), A10l(lateral area 10), A44d(dorsal area 44), IFS(inferior frontal sulcus), A45c(caudal area 45), A45r(rostral area 45), A44op(opercular area 44), A44v(ventral area 44), A14m(medial area 14), A12/47o(opercular area 12/47), A11l(lateral area 11), A11m(medial area 11), A13(area 13), A12/47l(lateral area 12/47), A38m(medial area 38), A41/42(area 41/42), TE(TE1.0 and TE 1.2), A22c(caudal area 22), A38l(lateral area 38), A22r(rostral area 22), A21c(caudal area 21), A21r(rostral area 21), A37dl(dorsolateral area 37), aSTS(anterior superior temporal sulcus))

**
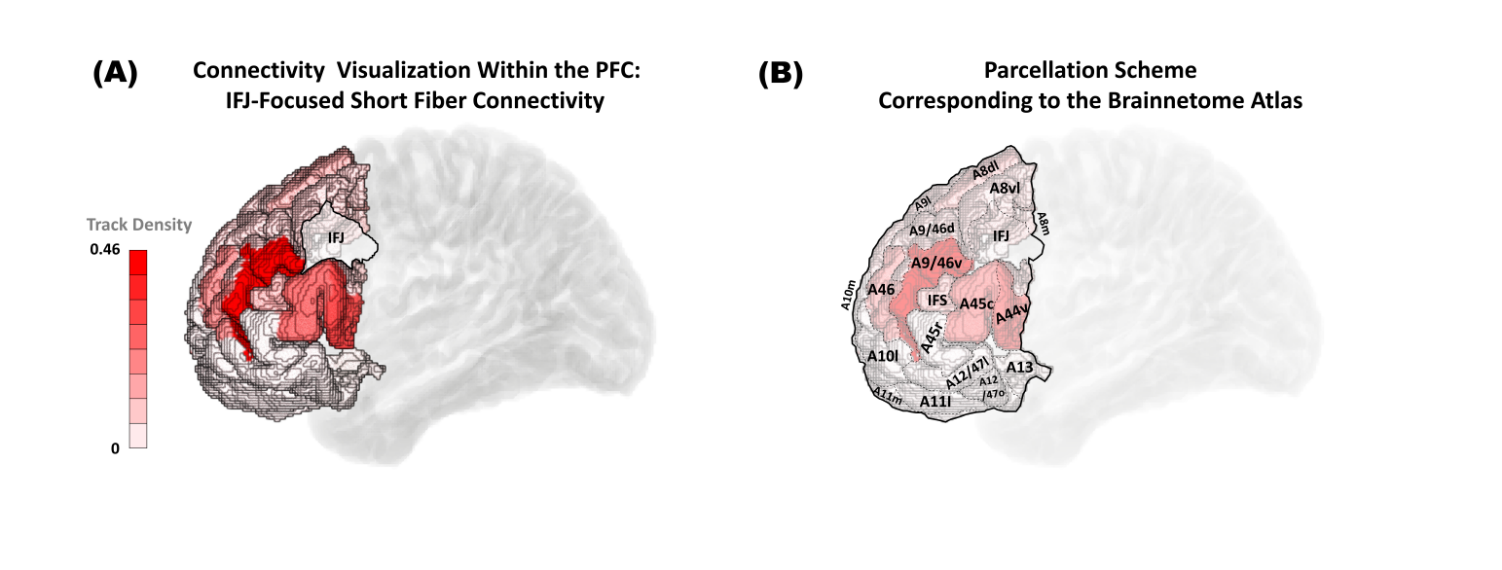
**

**Supplementary Figure 3. [A]** Connectivity visualization within the PFC: IFJ focused short fiber connectivity **[B]** Parcellation scheme corresponding to the Brainnetome atlas.

(**Abbreviation**: A8m(medial area 8), A8dl(dorsolateral area 8), A9l(lateral area 9), A9m(medial area 9), A10m(medial area 10), A9/46d(dorsal area 9/46), IFJ(inferior frontal junction), A46(area 46), A8vl(ventrolateral area 8), A10l(lateral area 10), A44d(dorsal area 44), IFS(inferior frontal sulcus), A45c(caudal area 45), A45r(rostral area 45), A44op(opercular area 44), A44v(ventral area 44), A14m(medial area 14), A12/47o(opercular area 12/47), A11l(lateral area 11), A11m(medial area 11), A13(area 13), A12/47l(lateral area 12/47))

**Supplementary Table 1.** Track density percentile for the PFC subregions regarding the PFC-IPC connectivity.

**Supplementary Table 2.** Track density percentile for the PFC subregions regarding the PFC-TC connectivity.
